# Translational Informatics for Parkinson’s Disease: from Big Biomedical Data to Small Actionable Alterations

**DOI:** 10.1016/j.gpb.2018.10.007

**Published:** 2019-11-28

**Authors:** Bairong Shen, Yuxin Lin, Cheng Bi, Shengrong Zhou, Zhongchen Bai, Guangmin Zheng, Jing Zhou

**Affiliations:** 1 Institutes for Systems Genetics, West China Hospital, Sichuan University, Chengdu 610041, China; 2 Center for Systems Biology, Soochow University, Suzhou 215006, China; 3 Center for Translational Biomedical Informatics, Guizhou University School of Medicine, Guiyang 550025, China

**Keywords:** Parkinson's disease, Healthcare, Disease biomarker, Translational informatics, Systems modelling

## Abstract

**Parkinson's disease** (PD) is a common neurological disease in elderly people, and its morbidity and mortality are increasing with the advent of global ageing. The traditional paradigm of moving from small data to big data in biomedical research is shifting toward big data-based identification of small actionable alterations. To highlight the use of big data for precision PD medicine, we review PD big data and informatics for the translation of basic PD research to clinical applications. We emphasize some key findings in clinically actionable changes, such as susceptibility genetic variations for PD risk population screening, biomarkers for the diagnosis and stratification of PD patients, risk factors for PD, and lifestyles for the prevention of PD*.* The challenges associated with the collection, storage, and modelling of diverse big data for PD precision medicine and **healthcare** are also summarized. Future perspectives on **systems modelling** and intelligent medicine for PD monitoring, diagnosis, treatment, and healthcare are discussed in the end.

## Introduction

The disease spectrum is changing with the ballooning of elderly society. The morbidity and mortality of geriatric disease, including Alzheimer’s disease (AD) and Parkinson's disease (PD), are increasing globally [1]. The social burden of the care of elderly patients is becoming a considerable challenge because of the lack of sufficient medical and labour resources. The shortage of medical care resources and the increasing demand of the ageing society will be obstacles to social and economic development.

PD is one of the most common neurodegenerative diseases (NDDs) in elderly people. As the most frequent movement disorder, PD usually develops very slowly, although it can be accelerated in the latter years. It can take more than 20 years to proceed the beginning of neurodegeneration to the appearance of prodromal symptoms *en route* and to the manifestation of typical clinical symptoms of PD [2]. A search of the PubMed database with the term “Parkinson’s disease [tiab] OR Parkinson disease [tiab]” retrieves more than 87,500 records of PD studies at present. Nonetheless, the causative and molecular mechanism of PD remains elusive, although it is generally believed to involve complex interactions between genetics [3], gut microbiota [4], environmental factors [5], as well as unhealthy lifestyles [6]. These complex interactions pose great challenges in gaining a comprehensive understanding of the holistic mechanism underlying PD pathogenesis and progression.

Early diagnosis and prevention of PD is preferred over late clinical treatment of the disease because it can alleviate both social demand and family burden. Many basic questions remain to be addressed for PD studies before potential translation, such as the identification of biomarkers for personalized diagnosis and stratification of patients [7], the discovery of genetic or environmental factors for the screening of highly susceptible populations, and the finding of a positive lifestyle to facilitate personalized healthcare of elderly people [8], [9]*.* To investigate the molecular mechanisms underlying PD and answer the aforementioned questions, sufficient data and information about the genotypes and clinical phenotypes of different subtypes of PD are prerequisite to model the complex interactions.

**Figure 1:**
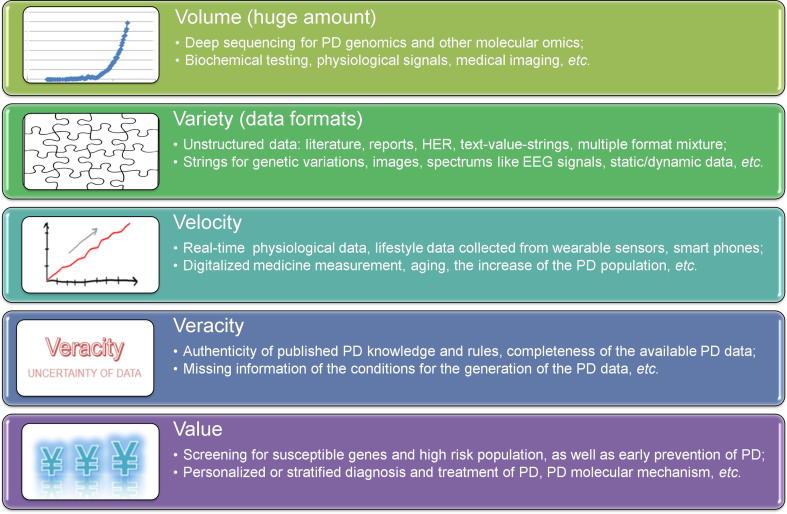
**The 5 Vs of PD big data** PD, Parkinson’s disease; EHR, electronic health record; EEG, electroencephalograph.

In recent decades, we have witnessed a rapid development of biotechnologies, especially high-throughput sequencing technologies. Deep sequencing for genetic architecture, gene expression and epigenetic patterns is becoming less expensive, and the costs of whole-genome sequencing have decreased from hundreds of million dollars to hundreds of dollars. Not only has the sequencing data accumulated at an unprecedented rate, the physiological data collected from different wearable sensors, the biochemical data detected by point-of-care tests, and the medical imaging data, including magnetic resonance imaging (MRI) and positron emission tomography computed tomography (PET-CT), are also increasing rapidly. We are now in a big data and digital medicine era [10], [11]. Data from healthy people as well as preclinical and clinical data from patients together contribute to the big volume of big PD data for future data-driven medicine (Figure 1).

The 5 Vs of big data characterization for PD are shown in Figure 1. The data formats vary and include strings for genetic data, images for PD brain structures, unstructured or semi-structured formats with real values, text for electronic health record (EHR), and time series data for physiological signals. The digitalization of diverse measurements speeds up the generation of all kinds of PD data. In particular, wearable sensors combined with smart phones make it possible to collect data real-time and obtain dynamic electroencephalograph (EEG). These would help with the monitoring and diagnosis of PD patients [12]. The identification of actionable key players and alterations from the considerably large, noisy and diverse unstructured big data is the goal for translational informatics studies. In this review, we mainly discuss the value of PD big data mining, as well as the challenges and perspectives for the translation of PD big data to valuable biomarker discovery and risk factor screening for the future clinical management and healthcare of PD.

## Actionable alterations for PD diagnosis and prevention

The traditional paradigm of translational research for disease biomarker or risk factor discovery is often from small data to big data. It starts from a hypothesis-driven investigation of the biological functions of few genes, proteins, or other biological molecules, followed by test of their biological functions and medical roles, moving from cell lines, animal models, and a small number of patients to big population validation. Biomarker and/or drug discoveries often fail in last-phase trials because the features or discoveries obtained from small data do not always work well in a big and diverse data space.

**Figure 2:**
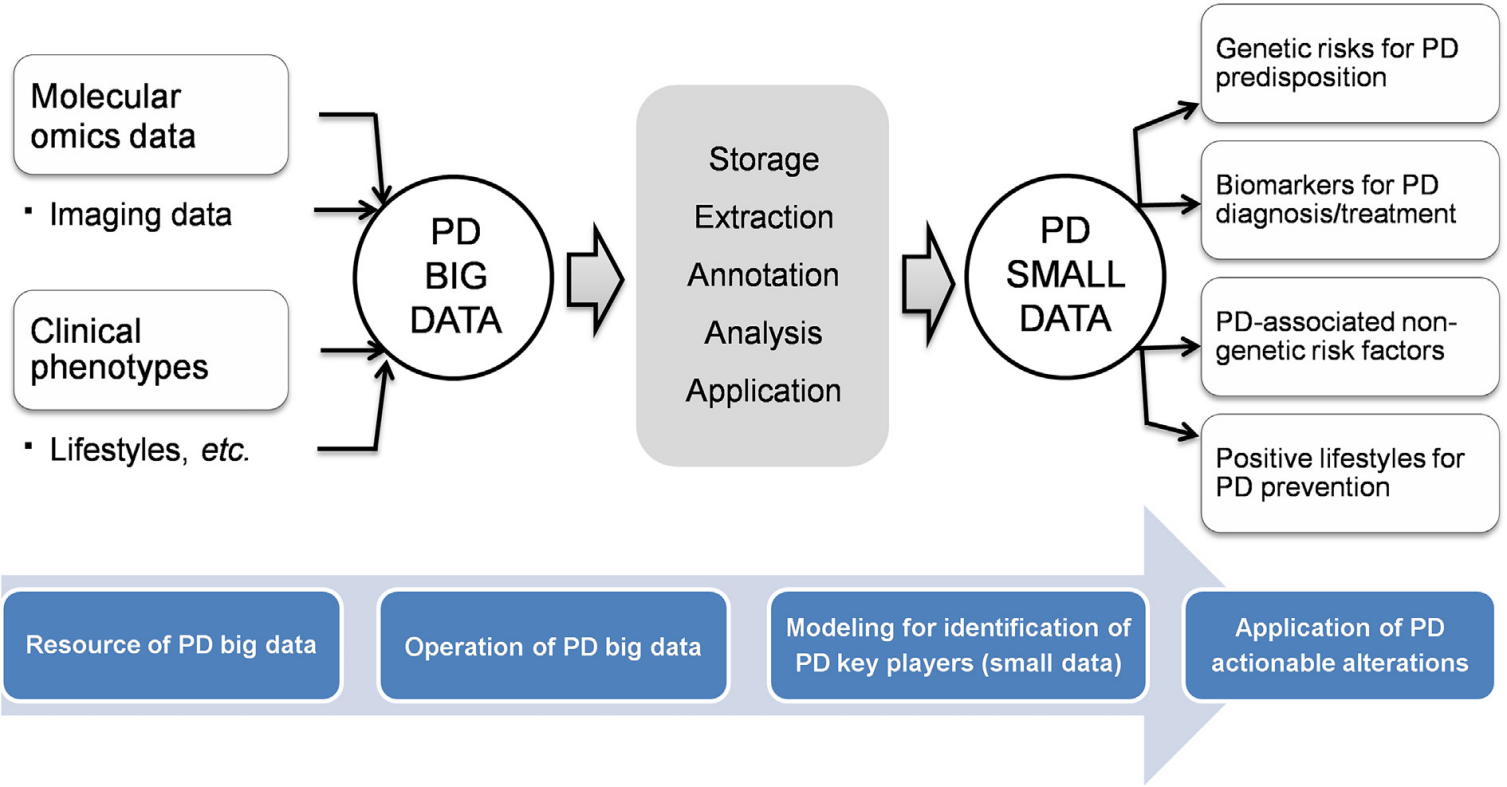
**PD translational informatics: from big data to small alterations**

Nowadays, the paradigm of biomedical research is shifting to one involving a move from big data to small data. Identifying small but important actionable alterations from big data mining and systems biological modelling is becoming possible. In this section, we discuss the clinically actionable alterations from four aspects. These include PD susceptibility genetic variants, biomarkers for PD diagnosis and prognosis, non-genetic PD risk factors, and lifestyles positively or negatively affecting PD (Figure 2).

### Genetic alterations and susceptibility to PD

Before 1997, when mutations in *SNCA*, the gene encoding synuclein alpha, were screened from PD families, PD was recognized as a sporadic and typical non-genetic disorder [13]. With the increasing number of genetic risks identified, PD is now considered a disorder ranging from monogenic to polygenic inheritance associated with a complex interaction between genetics, lifestyle, and environmental exposures [14].

Table 1 provides a partial lists of previously reported genetic risk factors for familial or sporadic PD, covering single-nucleotide polymorphisms (SNPs), haplotypes, copy number variations, and other polymorphisms. In our Neurodegenerative Disease Variation Database (NDDVD), more than 600 variants in 43 genes associated with PD have been collected [14]. Some of the genetic variants could be driver mutations, such as mutations in *SNCA* and *LRRK2. LRRK2* is a key player and a common inheritable factor in PD. It encodes leucine rich repeat kinase 2, a kinase involved in the signalling pathways related to neuronal death, and could be a potential therapeutic target for PD [15], [16], [17]. While other variants are mostly passenger but not driver mutations, they can work together to disrupt the biological system and cause PD [18]. At present, both the common disease-multiple rare variant (CDMV) and common disease-common variant (CDCV) hypotheses could be applied to explain the genetic variants for PD [19], and the penetrance of each gene variant may be associated with different populations, ages, genders, ethnicities, *etc.* Most of the complex cases cannot be reasonably explained yet. The cumulative effects of the variants on PD need to be evaluated on an individual basis. Two challenges remain for the genetic study of PD. The first is the discovery and curation of more variants, as each of them may have only a small effect on the pathogenesis and progression of PD, and the second is the building of models to accurately predict the cumulative effect of these genetic variants.

**Table 1:** **PD genetic risk factors**




*Note*: Variants were mentioned in different formats in previous publications and renamed in this article per the recommendation of Human Genome Variation Society (HGVS) for consistency. PD, Parkinson's disease; SNP, single nucleotide polymorphism; CNV, copy number variation; fs, frame shift; IVS, intervening sequence.

### PD-associated non-genetic risk factors

Although genetic factors could be important or even act as the driving force behind the pathogenesis and progression of PD, genetic susceptibility can explain only a small portion of PD cases. Many non-genetic factors are found to increase the risk of PD. Table 2 lists the previously identified PD-associated epidemiological and environmental factors. Gender and age, especially the maternal age, are known epidemiological factors that are significantly associated with PD. However, the association is conditional and could vary between individuals, given the contradictory reports of previous studies [20], [21]. Many psychiatric disorders can also cause PD, such as anxiety and depression [22], [23], [24]. Since the different systems in our body interact and are linked with each other, many diseases in other systems, such as cardiovascular disorders and metabolic syndrome, can also be comorbidities or complications of PD, as listed in Table 2, and therefore risk factors for PD. Additionally, environmental exposure to pesticides, chemical solvents, drugs, and virus infections can affect gene expression and even the ecological distribution of gut microbiota [25]. The complex interactions between these factors form a network that together regulates our biological systems and determines the course of PD.

**Table 2:** **Epidemiological and environmental risk factors for PD**

More genetic and non-genetic risk factors are expected to be identified in future screening. However, their contribution to the pathogenesis of PD is too complex to investigate without big data collected from PD patients and healthy people as controls. Two complementary approaches are now available for the screening of PD risk factors or biomarkers. One approach is the cross-sectional cohort study designed to collect medical data from a group of people and then to identify statistically significant features of disease risk factors [26], [27], while the other approach is the longitudinal personalized study of individuals to identify patterns associated with disease and health status among these individuals [28]. The former approach can identify important features common to a population, but a one-size-fits-all threshold may not be accurate for individuals when applying statistically averaged indicators to disease diagnosis, like applying the same blood pressure thresholds to the diagnosis of high hypertension. The latter approach assesses the individual’s health status based on personalized reference data, which could be more accurate than the former approach for a personalized diagnosis, although more longitudinal data on individuals need to be collected for the latter approach.

### Lifestyle changes for the prevention of Parkinson’s disease

Compared to genetic and environmental factors, lifestyle can be adjusted more easily for the prevention of disease and improvement of health. As shown in Table 3, two types of lifestyle behaviours have been found to positively and negatively affect PD. Although smoking is a risk factor for cancer, especially lung cancer, it could be a preventive factor for PD [29], [30]. In addition, the consumption of coffee, tea, wine, *etc*. could be helpful for the prevention of PD as well. Although the relationships between lifestyles and diseases are complicated by their interaction with genetic and environmental factors, negative lifestyle behaviours should be adjusted to reduce the risk of PD. Particularly, in the era of elderly society, actively changing lifestyles for the prevention of disease is a better strategy for healthcare than traditional clinical intervention, which is cost prohibitive and requires more labour and medical resources. Lifestyle management for high-risk populations is an efficient way to prevent PD [31].

**Table 3:** **Positive and negative lifestyles for PD**

To unravel relationships between lifestyle and disease prevention, exclusive use of biomedical data is inadequate, and mining data from social networks will be important. Differences between “translational bioinformatics”, “translational biomedical informatics”, and “translational informatics” are related to the data types analyzed. Bioinformatics generally focuses on data at the molecular level, such as genome, transcriptome, proteome, and metabolome data, whereas biomedical informatics also involves cell/tissue imaging data, patient data, and the public health data. As noted, translational informatics will cover a wider scope of data relative to the other two methodologies, as it includes not only biomedical data but also social network data associated with lifestyle information.

### Driver player and biomarker discovery for personalized medicine

Biomarkers are a class of indicators that are able to predict changes in biological systems and provide specific signatures for disease diagnosis, prognosis, or treatment [7]. In recent decades, many PD-related biomarkers, including molecules, images, clinical symptoms, and physiology, have been identified for monitoring the occurrence and progression of this complex disease.

As illustrated in Table 4, biological molecules, such as genes, RNAs, proteins, and metabolites, play important roles in PD evolution. For example, cerebro-spinal fluid (CSF) *α*-synuclein was one of the well-studied proteins implicated in PD pathogenesis, and its genetic variability was a prognostic marker for PD, PD with dementia, and dementia with Lewy bodies [32]. Ritz et al. [33] demonstrated that *α*-synuclein genetic variants were associated with the development of faster motor symptoms in idiopathic PD. In addition, Ballard et al. [34] reported that CSF *α*-synuclein had the potential for diagnosing PD and related dementias. Mollenhauer et al. [26] found that CSF *α*-synuclein was also a useful indicator in PD patients undergoing dopamine replacement therapy. Moreover, plasma and skin nerve *α*-synuclein is valuable in predicting PD cognitive impairment and idiopathic PD, respectively [35], [36]. Another key player, CSF *β*-amyloid 1–42, was a powerful predictor of the progression of cognitive impairment, dementia, and dopa-resistant gait in PD. For example, a lower level of CSF *β*-amyloid 1–42 was common in advanced PD patients with cognitive decline and could be used to predict cognitive impairment in newly diagnosed PD [37]. Alves et al. [38] indicated that the CSF levels of *β*-amyloid 1–42 were lower in PD patients with dementia. The abnormal expression of this protein increased the risk of dementia development, which was used for the early prognosis of PD dementia [38]. Moreover, a decrease in *β*-amyloid 1–42 was also involved in the pathology of dopa-resistant gait in early PD [39].

**Table 4:** **Literature-reported biomarkers for diagnosis, prognosis, and treatment of PD**




*Note*: Molecule types includes gene, RNA, protein, and metabolite; CSF, cerebrospinal fluid; PET, positron emission tomography; EEG, electroencephalograph; SPECT, single photon emission computed tomography; REM, rapid eye movement.

In contrast to molecular biomarkers, imaging and clinical symptoms are often directly used clinically for PD investigation. With the development of medical imaging techniques, PET imaging, quantitative EEG, and single photon emission computed tomography (SPE-CT) have been widely used to screen key signatures to predict the progression of dementia, the severity of fatigue, and dopaminergic responsiveness in PD patients [40], [41], [42], [43]. Clinical symptoms, on the other hand, call attention to an early diagnosis of PD and its associated phenotypes. For example, episodic anxiety was found to be more specific for the anxiety subtypes in PD than the persistent anxiety. Episodic anxiety was a significant factor related to PD severity and duration [44]. Based on a case-control study, Pradhan et al. [45] uncovered that characteristics in grip force modulation, *e.g.*, force and movement quality, were sensitive measurements in detecting early PD and tracking the clinical progression of PD patients. A circadian change in core body temperature, *i.e.*, rectal temperature, was detected in PD patients with depression, suggesting its possibility in predicting PD depression [46]. Typical clinical symptoms could be evaluated for PD prognosis tracking in addition to diagnosis. Willis et al. [47] showed that dementia was a prevalent trait in PD patients, which strongly affected the survival of PD patients and could increase the chance of mortality. In addition to clinical symptoms, some physiological features were connected with the functional alterations observed in PD. For example, the length of the electrophysiologic subthalamic nucleus and the connectivity between the stimulation site and subthalamic nucleus could predict the outcome of deep brain stimulation in PD [48], [49]. Brain volume or thickness could also be used as a parameter to recognize cognitive impairment during PD development [50].

With the coming age of big data and digitalized medicine, more novel and important functional components in our biological systems will be discovered, such as how gut flora dysbiosis can affect brain function and how it is associated with PD through the microbiota-gut-brain axis. Therefore gut flora dysbiosis can be used as a new type of biomarker for PD [51].

## Data integration and modelling for translational informatics of PD

### PD biomedical data standardization and integration

Big PD biomedical data are diverse and could include the following data types. (1) The first one is different omics data, such as genomic data on genetic structure, variant susceptibility to PD, and the transcriptomic, as well as proteomic and metabolomic data characterization of the abnormal states of PD. (2) The second is neuroimaging data, such as MRI for brain functional structure, as well as PET-CT and SPE-CT for altered brain structures in PD patients. (3) The third type of data are physiological signals, including EEG and electrocardiography (ECG) to reflect PD patients’ cognitive impairment or other clinical features [52], [53]. (4) The fourth type of data are information from EHR or electronic medical records (EMR). These include patient’s demographic data, results of clinical laboratory tests, medical history, use of specific medications, and other clinical phenotype data. (5) Finally, the last type of data are epidemiological data on lifestyle, environment, or social network information.

**Figure 3:**
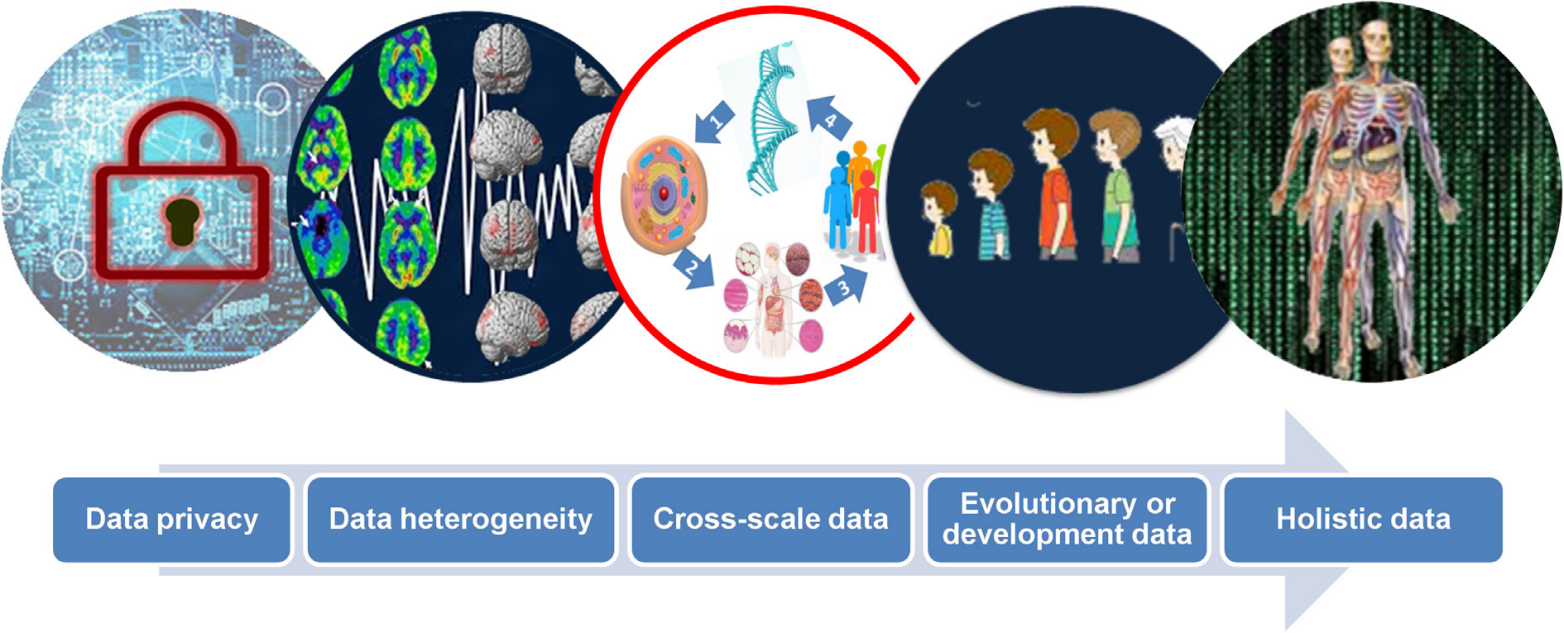
**Diverse data types and big data challenges**

These big PD biomedical data could be static or dynamic and can reflect the development of PD from early prodromal symptoms to the latter clinical stages. As presented in Figure 3, big PD data have several characteristics that differ from those of other types of big data such as business, market, and social network data. Notably, data privacy is important for patients ethically, and the data need to be transformed before they can be accessed by users and researchers. The heterogeneity of PD is caused by the interaction between multiple pathogenic factors, such as genetics, lifestyle, and environment. In addition, these data could be collected from different platforms and stages of different patients. The PD data listed above could be collected at different levels ranging from molecular to cellular, tissue, or individual levels at different time points. These properties make standardization and integration very challenging. The challenges for PD data integration could include the following three aspects.

#### Challenge 1: diversity, standardization, and sharing of big PD biomedical data

The genesis and progression of PD is caused by the complex interactions between genetics, environmental factors and lifestyles and the PD phenotypes are therefore very diverse and heterogeneous [54], [55]. To understand mechanism of PD at the systems biological level, the different omics data need to be integrated and standardized for sharing and modelling. For the sharing of big PD biomedical data, two issues need to be considered. The first issue is data privacy preservation [56], [57], [58], [59]. Although many algorithms have been developed for the protection of patient’s genome information, further efforts are needed to preserve the patients’ information at the phenotype and family levels [60], [61], [62], [63]. The second issue is the development of ontology for the standardization of PD data, which could classify and standardize the PD specific concepts and synonyms and promote the sharing and integration of big biomedical data on PD.

#### Challenge 2: databases for big PD biomedical data and knowledge

A database and a knowledge base for diverse PD data are needed for the modelling and understanding of the pathogenesis and progression of PD. Table 5 lists the existing PD databases. PDGene is a comprehensive online resource of potential risk loci in PD [64]. After data from all the published articles and genome-wide association studies (GWAS) were extracted, deep meta-analyses were performed on millions of polymorphisms from different GWAS datasets or PD-related studies. A total of 11 loci, *e.g.*, *GBA*, *LRRK2*, *MAPT*, *PARK16*, and *SNCA*, were significant genome-wide for PD risk evaluation. ParkDB is another database aimed at recording key molecular events during PD development [65]. It contains a large number of re-analysed and annotated microarray datasets, which are advantageous for screening expression signatures associated with PD under different biological backgrounds. PDmutDB is a PD mutation database that comprises information on all known mutations in the genes associated with PD development [66]. Through expressed sequenced tags (ESTs) on substantia nigra tissues from healthy and PD populations, PDbase was built to capture PD-related genes and genetic variations [67]. In addition, the database integrated several valuable resources for PD annotation and provides information such as mitochondrion proteins, microRNA-gene regulations, structural variations in PD-related genes, and pathways/networks within protein–protein interactions to better understand the causes of PD. An international and multi-centre study, PPMI, also collected diverse data from PD patients for future biomarker discovery and personalized PD therapy [68]. Although many databases have been established, with more digital data from PD patients and related resources available, we could collect big PD biomedical data, especially PD-associated phenotype data, to conduct studies to obtain a holistic description and mechanism of PD.

**Table 5:** **The currently-available PD databases**

#### Challenge 3: cross-level and dynamic integration of PD biomedical data

Many levels exist between genotype and PD clinical symptom phenotype, such as the molecular phenotype and cellular phenotype, and physiological signals could also be a type of phenotype. Therefore, the relationship between genotype and a patient’s clinical phenotype is very complex. At present, most of the PD data and information at all these levels are isolated from each other and need to be interlinked and integrated. In the time dimension, these data can be ordered based on pathogenesis and progression. Traditionally, data at these different levels are often statistically averaged and reasoned for correlation studies; however, these methods often average the patterns in subgroups of the studied samples. Paired data for all the levels between the genotype and disease phenotype will be essential to the precision modelling of the disease systems, and if the paired data are collected in a time series, then the PD progression and trajectory could be modelled. The Cancer Genome Atlas (TCGA) for cancer research is a typical paradigm that could be applied to PD data integration in the future to obtain cross-level and dynamic integration of PD data.

### Big PD data mining and modelling for translational application

**Figure 4:**
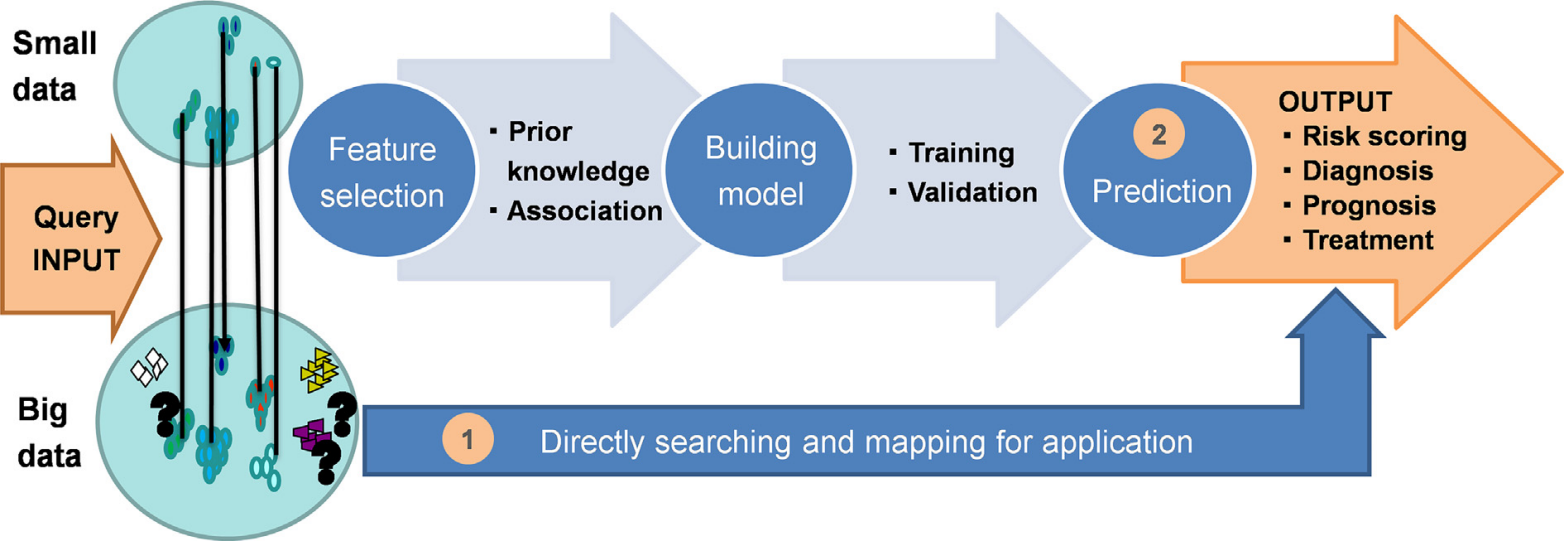
**Big data model for precision prediction**

As shown in Figure 4, when small data are used for PD modelling, some complex patterns cannot be represented in this small data space; thus, when a model trained from a small data set is applied to a big data space, the model will unlikely be successful. With big PD biomedical data available, we will have the chance to use these data to model and mine the knowledge and patterns hidden in these big data, and some questions that could not be answered before could now be investigated. The following three modelling challenges are expected when translating big PD biomedical data to clinical application.

#### Challenge 4: holistic and systems-level modelling and mechanism-based key player identification

Without sufficient data for modelling PD pathogenesis and progression, we can only partly address the complex PD “elephant”. Holistic and systems-level characterization of the PD mechanism is necessary to understand its complexity and heterogeneity. The systems-level identification of key players, such as biomarkers for classifying PD and risk factors for high-risk population screening, will be a challenge for future PD translational informatics. Compared to traditional disease-gene recognition, systems-level discovery of genes, pathways, modules or sub-networks that drive systems to change from a healthy to a disease state will be the objectives of big data-based modelling.

Table 6 lists previously reported PD-associated pathways, as based on our experience in cancer research, it is often easier to find common pathways for complex diseases than to find common disease genes [69], [70].

**Table 6:** **Biological pathways associated with PD pathogenesis and molecular mechanisms**




*Note*: DBL, 3,4-dihydroxybenzalacetone; MPTP, 1-methyl-4-phenyl-1,2,4,6,-tetrahydropyridine; 6-OHDA, 6-hydroxydopamine; MPP, 1-methyl-4-phenylpyridinium.

#### Challenge 5: modelling of PD dynamic progression and systems-level control of PD progression

Since complex PD is the product of a dynamic interaction between the patient’s genetics, environment and lifestyle, the cause and course of PD are dynamically changed. With dynamic information from the human body, such as routine blood testing and the real-time collection of physiological signals [12], modelling of the dynamic evolution of PD is possible, and the identification of the key hubs and connections in these dynamic systems will be a challenge but opportunity for rational drug design or lifestyle changes to control the development of PD [71], [72].

#### Challenge 6: general rule discovery for basic research and prevention of PD

Big data make artificial intelligence, including deep learning and reinforcement learning, applicable to the analysis of big data and PD studies [73], [74]. Furthermore, knowledge of PD is accumulating and could be used to improve predictions [75]. However, the discovery of general rules for the molecular mechanism of disease is still very necessary to study complex systems. Our previous study discovered the rich-get-richer rule for a new gene’s functional evolution [76], and for disease progression and prevention, the discovery of general rules from big biomedical data will be a complementary objective to personalized and precision PD medicine.

We face two additional challenges in the translational application of findings from big data to PD clinical management and healthcare.

#### Challenge 7: screening of populations at high risk of PD

Integrating genetic susceptibility and environmental and lifestyle factors together to build a systems model for the precision screening of populations at high risk for PD will be essential for the early prevention and intervention of PD.

#### Challenge 8: PD sub-population searching for personalized treatments

PD patients can be treated with levodopa or other dopamine replacement drugs, and surgical approaches, including pallidotomy and thalamotomy, could be alternatives. The responses and side effects of these therapies can be personalized to each patient [77], [78]. Big data could also provide a direct search and mapping method for the clinical decision of which treatment to use, as shown in Figure 4. However, identifying suitable subpopulations for efficient treatments is always a challenge for clinical application.

## Perspectives on future translational PD informatics

The driving forces for the translational informatics study of PD come from technological, scientific and social aspects. Technically, genome variants, gene expression and epigenetic alterations, *etc.* could be measured by advanced next-generation sequencing technologies. Clinical laboratory tests may be easily performed by point-of-care tests in a direct-to-consumer mode. Physiological signals could be detected in real time by the combination of wearable sensors, smart phones and cloud computing, and everyone, including healthy people, family members, nurses, medical doctors and data analysts, could be linked via the internet in the cloud to manage the data in a crowdsourcing model.

**Figure 5:**
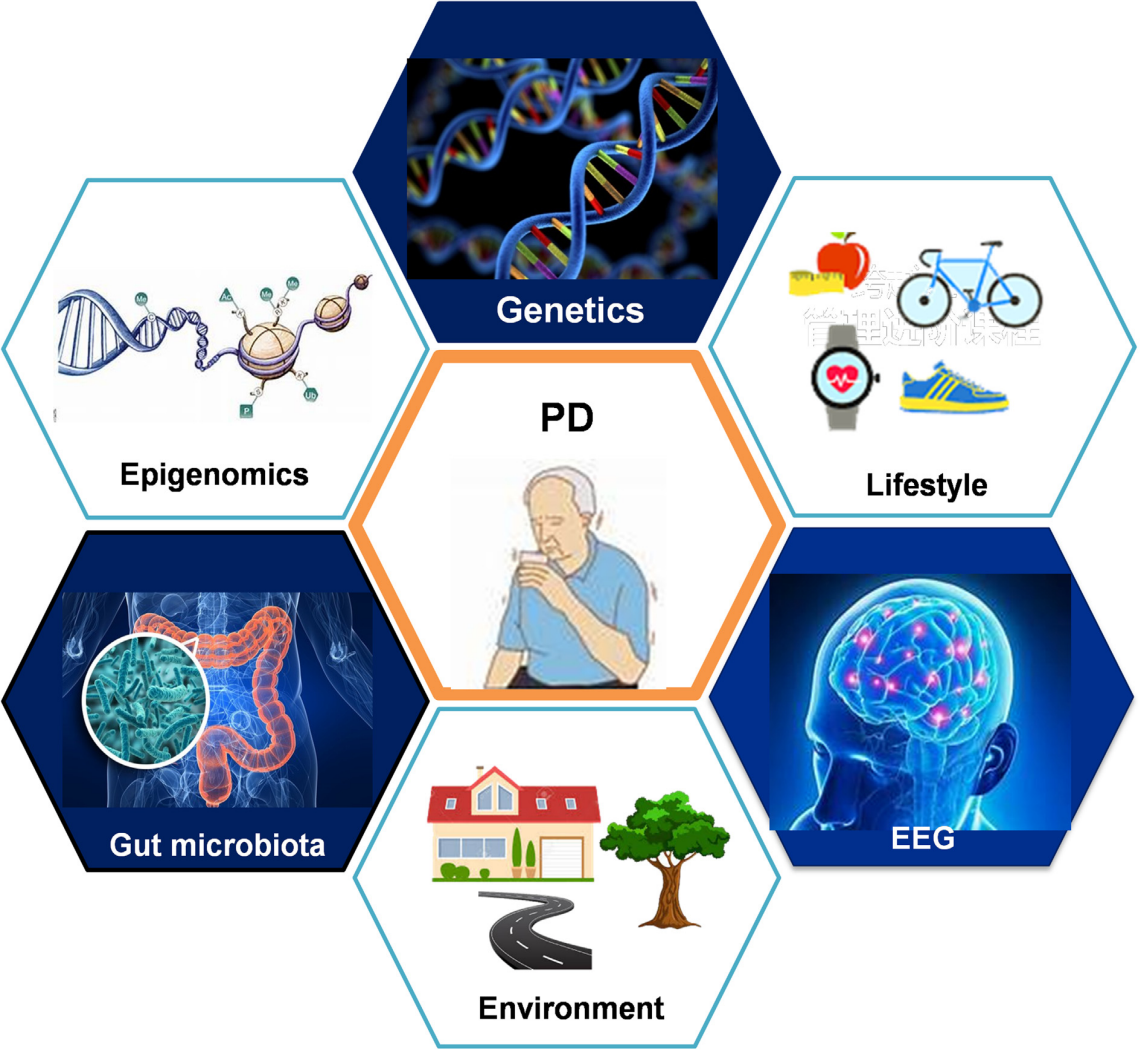
**From personalized data to systems healthcare of PD patients**

Scientifically, the interactions between genetics, lifestyle and physiological signals as well as the microbiota and the environment are deepening our knowledge and understanding of PD (Figure 5). Systems biology and evolutionary medicine-level modelling of these interactions are becoming the paradigm to investigate complex diseases such as cancer and NDDs, including PD. Recent genetic editing methods, findings regarding brain-gut connections and studies on the diverse molecular mechanisms of PD pathogenesis all accelerate basic PD research discoveries for clinical applications.

Regarding the social and economic aspects, the ageing society and the considerable cost of the clinical management of PD urgently demand improved prevention and prediction of PD, and all governments are promoting the market of healthcare, especially for senile diseases such as AD and PD*.* By addressing the three challenges to PD data integration described above, translational informatics for PD studies will have considerable opportunities for scientific discovery and healthcare applications.

## Competing interests

The authors have declared no competing interests.
